# Does task complexity impact the neurovascular coupling response similarly between males and females?

**DOI:** 10.14814/phy2.15020

**Published:** 2021-09-13

**Authors:** Joel S. Burma, Rebecca M. Wassmuth, Courtney M. Kennedy, Lauren N. Miutz, Kailey T. Newel, Joseph Carere, Jonathan D. Smirl

**Affiliations:** ^1^ Cerebrovascular Concussion Lab Faculty of Kinesiology University of Calgary Alberta Canada; ^2^ Sport Injury Prevention Research Centre Faculty of Kinesiology University of Calgary Calgary Alberta Canada; ^3^ Hotchkiss Brain Institute University of Calgary Calgary Alberta Canada; ^4^ Integrated Concussion Research Program University of Calgary Calgary AB Canada; ^5^ Alberta Children’s Hospital Research Institute University of Calgary Calgary Alberta Canada; ^6^ Human Performance Laboratory Faculty of Kinesiology University of Calgary Calgary Alberta Canada; ^7^ Libin Cardiovascular Institute of Alberta University of Calgary Alberta Canada; ^8^ Faculty of Health and Exercise Science University of British Columbia Kelowna BC Canada

**Keywords:** neurovascular coupling, posterior cerebral artery, simple shapes, transcranial Doppler ultrasound, Where's Waldo?

## Abstract

**Background:**

While previous studies have demonstrated a complex visual scene search elicits a robust neurovascular coupling (NVC) response, it is unknown how the duration of visual stimuli presentation influences NVC metrics. This study examined how stimuli duration, in addition to biological sex and self‐reported engagement impact NVC responses.

**Methods:**

Participants (n = 20, female = 10) completed four visual paradigms. Three involved simple visual shapes presented at 0.5‐, 2‐, and 4‐s intervals in randomized orders. The fourth paradigm was a complex visual scene search (“*Where's Waldo?*”). Participants completed eight cycles of 20‐s eyes‐closed followed by 40‐s eyes‐open. Transcranial Doppler ultrasound indexed posterior and middle cerebral artery velocities (PCA and MCA). Participants self‐reported their engagement following each task (1 [minimal] to 10 [maximal]).

**Results:**

The “*Where's Waldo?*” task evoked greater PCA percent increase (all *p* < 0.001) and area under the curve during the first 30‐s of the task (all *p* < 0.001) compared to simple shapes. Females displayed greater absolute baseline and peak PCA and MCA velocities across all tasks (all *p* < 0.002). Subjective engagement displayed moderate correlation levels with PCA percent increase (Spearman *ρ* = 0.58) and area under the curve (Spearman *ρ* = 0.60) metrics in males, whereas these were weak for females (Spearman *ρ* = 0.43 and *ρ* = 0.38, respectively).

**Conclusions:**

The complex visual paradigm “*Where's Waldo?*” greatly augmented the signal‐to‐noise ratio within the PCA aspects of the NVC response compared to simple shapes. While both sexes had similar NVC responses, task engagement was more related to NVC metrics in males compared to females. Therefore, future NVC investigations should consider task engagement when designing studies.

## Graphical abstract

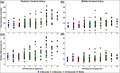

**New & Noteworthy**

A simple shapes task elicited a modest neurovascular coupling (NVC) response, regardless of image presentation duration (4, 2, or 0.5 s). Conversely, a complex “*Where's Waldo?*” search produced a robust response that maximized the NVC response. While absolute cerebral blood velocity metrics were higher in females, no differences were found between biological sexes regarding the NVC response. However, subjective task engagement had a greater correlation within males, highlighting the need for researchers to use more engaging tasks for males to elicit a similar NVC response compared to females.

## 1 INTRODUCTION

The cerebrovascular regulatory system has several mechanisms that function to maintain the adequate delivery of nutrients and oxygen to the brain (Willie et al., 2014). These include: neural activity, blood pressure, arterial oxygen and carbon dioxide alterations, sympathetic nervous activity, and cardiac output (Willie et al., 2014). Neurovascular coupling (NVC) describes the phenomena where blood supply is increased within active regions of the brain, which also enables the removal of associated metabolic by‐products (Phillips et al., 2016). This coupling between neurons and the vasculature is an important and extensive physiological process that ensures the working cortical regions of the brain are adequately perfused to perform a given task (Phillips et al., 2016). The NVC response has been shown to be impaired following a plethora of clinical conditions (e.g., spinal cord injury (Phillips et al., 2014), traumatic brain injury (Hinzman et al., 2014), Alzheimer's disease (Nicolakakis & Hamel, 2011), etc.). Therefore, it is imperative clinical investigations utilize the most robust methodological approaches to delineate between healthy and clinical populations.

Across the literature, the NVC response has been assessed using a variety of different techniques and methods (Iadecola, 2017). For example, a previous study compared how visual stimuli of differing complexity impacted the NVC response when using transcranial Doppler ultrasound (TCD) (Smirl et al., 2016). Such stimuli included viewing checkerboards or other simple shapes (low complexity), reading an article (moderate complexity), and a visual scene search (“*Where's Waldo?*”) (high complexity) (Smirl et al., 2016). The low and moderate complexity tasks only elicited a modest activation response within the posterior cerebral artery (PCA) (Smirl et al., 2016). Conversely, the “*Where's Waldo?*” enhanced the signal‐to‐noise ratio and resulted in a robust NVC response within both the total PCA activation (area under the curve) and the relative percent increase (Smirl et al., 2016). Moreover, since the visual search consists of many different objects, shapes, and colors, both the ventral (“*what*”) and dorsal (“*where*”) streams of visual perception are activated to a greater extent (Chang et al., 2014).

However, there are several methodological considerations that have yet to be fully elucidated with respect to assessing the NVC response. It is important to consider that both the low and moderate complexity tasks utilized within the previous study (Smirl et al., 2016) would have less contribution from the ventral visual processing stream compared to the complex task, as the same shape was repeatedly presented in a single color. Therefore, it remains unknown, the extent to which the “*Where's Waldo?*” task activated other regions of the brain supplied by the middle cerebral artery (MCA) or arteries that branch from it (e.g., Posterior parietal cortex [spatial attention], orbitofrontal cortex [object‐value categories], anterior/posterior inferotemporal cortex [view‐ and position‐invariant/selective categories]), respectively (Chang et al., 2014). Furthermore, previous research has only compared complex visual tasks and simple visual tasks, such as reading and viewing colored dots, which fail to induce saccades and fixation durations comparable to those presented during a complex search paradigm (Rayner, 1998, 2009; Rayner & Raney, 1996; Smirl et al., 2016). Therefore, a major contrast between the prior stimuli employed in the NVC field may be related to the manner in which the visual information is processed. Such differences in visual processing provide an indication for understanding how CBV parameters respond to finding simple targets that induce similar image search patterns often observed during complex visual tasks (Gitelman et al., 2002). Moreover, three primary regions involved in the control of eye movements (i.e., frontal eye field, supplementary eye field, and the dorsolateral prefrontal cortex) are also, in part, supplied by the MCA (Vernet et al., 2014). Therefore, it is fundamental to understand the extent the “*Where's Waldo?*” task activates the visual centers supplied by the PCA, compared to brain regions supplied by the MCA.

The second methodological consideration surrounds subjective engagement within a given task. Previous research has suggested tasks with heightened engagement will elicit increased saccadic eye movements (Gitelman et al., 2002). Therefore, as the "*Where's Waldo?*" task is goal‐oriented, it may increase someones relative engagement with task. Thus, it is unknown if the augmented NVC responses elicited from the “*Where's Waldo?*” task is due to eye movements or the nature of the task itself. This also highlights that subjective ratings of task engagement may play an underappreciated role regarding the NVC response. Nonetheless, there is a paucity of research examining how subjective self‐reported engagement within a task influences the NVC response.

Finally, a large limitation of previous cerebrovascular, cardiovascular, and exercise physiology research is the underrepresentation of females, as well as studies comparing between biological sexes (Norris et al., 2020). For example, there have been only a handful of studies that have included biological sex comparisons across different aspects of the aforementioned cerebrovascular regulatory mechanisms using robust methodology (Burma et al., 2020; Favre & Serrador, 2019; Tallon et al., 2020). This paucity of research warrants the need for future studies considering biological sex throughout the broader cerebrovascular regulatory mechanism literature, especially given the differences in hormonal concentrations among other factors (Blair, 2007). For example, previous research has highlighted females typically have greater CBV and cerebral blood flow compared to their male counterparts (Bertsch et al., 2009; Devous et al., 1986; Lu et al., 2011; Rodriguez et al., 1988). Others have suggested that higher levels of estrogen may increase the cerebral metabolic rate of glucose (Leenders et al., 1990; Marchal et al., 1992; Yamaguchi et al., 1986). Therefore, despite the different baseline values between males and females, it is unknown if greater estrogen levels may lead to a greater NVC response in females compared to their male counterparts.

This study built on a previous NVC methodological investigation (Smirl et al., 2016), which was aimed to address three primary research questions: (a) to understand how image presentation duration for simple visual stimuli affects the NVC response compared to the large PCA indexed NVC increase seen during the current standard complex visual scene search (“*Where's Waldo?*”); (b) to delineate if a correlation exists between self‐reported subjective ratings of engagement and the NVC response; and (c) to investigate potential sex differences within NVC metrics during similar tasks. It was hypothesized: (a) when comparing the simple visual searches, the quickest search task (0.5‐s intervals) would elicit the greatest CBV response within the PCA supplied regions of the brain, compared to the 2‐ and 4‐s paradigms; (b) the complex visual search task (“*Where's Waldo?*”) would evoke the greatest CBV response within the PCA supplied regions of the brain, compared to simple tasks designed to elicit similar saccadic eye patterns; (c) self‐reported measures of engagement would parallel CBV responses, suggesting a complex visual search would elicit the greatest CBV elevation and metrics of engagement; and (d) consistent with prior literature, females would have greater CBV and NVC metrics compared to their male counterparts, due to the aforementioned postulations.

## 2 MATERIALS AND METHODS

### 2.1 Ethical Approval

This study was approved by the Conjoint Health Research Ethics Board at the University of Calgary (REB20‐1662 and REB20‐2112) under larger studies examining the regulatory mechanisms of cerebral blood flow in humans. All subjects provided written informed consent and all procedures were followed in accordance with institutional guidelines.

### 2.2 Participants

A convenience sample of 39 healthy university students (22 females) were recruited to participate in the current investigation. Data collection took place in March, April, and July of 2021. Demographics for the participants are displayed in Table 1. Participants had no history of any cerebrovascular, neurological, cardiorespiratory, and/or musculoskeletal diseases. Participants were instructed to abstain from alcohol, caffeine, smoking, and vaping for a minimum of 8 hours prior to commencement of data collection. Additionally, participants refrained from exercise for at least 4 hours before data collection, as previous research has shown this to be a sufficient time frame for the recovery of NVC response (Burma et al., 2021). All protocols were thoroughly explained to participants and any questions were answered prior to the commencement of the study.

**TABLE 1:** Demographics of participants and testing conditions

	Total (n = 39)	Females (n = 22)	Males (n = 17)	Sex test statistic
Demographics				
Age (years)	23.6 ± 3.3	23.0 ± 3.2	24.5 ± 3.4	*p* = 0.173; *d* = 0.45
Height (cm)	169.9 ± 9.8	163.9 ± 5.5	178.8 ± 8.6	*p* < 0.001; *d* = 1.93
Weight (kg)	72.9 ± 16.0	65.4 ± 11.7	82.6 ± 15.9	*p* < 0.001; *d* = 1.23
BMI (kg/m^2^)	25.1 ± 3.7	24.3 ± 3.4	26.0 ± 4.1	p=0.144; *d* = 0.48
Environmental factors				
Barometric pressure (mmHg)	668.4 ± 3.9	668.3 ± 3.3	668.4 ± 4.7	*p* = 0.919; *d* = 0.03
Humidity (%)	30.9 ± 17.3	33.3 ± 17.9	29.2 ± 16.5	*p* = 0.471; *d* = 0.24
Temperature (°C)	21.5 ± 0.6	21.5 ± 0.6	21.6 ± 0.6	*p* = 0.452; *d* = 0.25
Forehead temperature (°C)	36.4 ± 0.6	36.5 ± 0.3	36.2 ± 0.8	*p* = 0.148; *d* = 0.45

Values are mean ± standard deviation. Independent *t*‐tests with Cohen's *d* were used to assess the differences in demographic and environmental factors between sex. Cohen's d effect size thresholds were <0.2 (negligible), 0.2–0.5 (small), 0.5–0.8 (moderate), and >0.8 (large). All demographic factors were measured at the beginning of each testing session. Centimeters (cm), kilograms (kg), body mass index (BMI), meters (m), millimeters of mercury (mmHg), percent (%), and degrees Celsius (°C).

### 2.3 Experimental protocols

**FIGURE 1:**
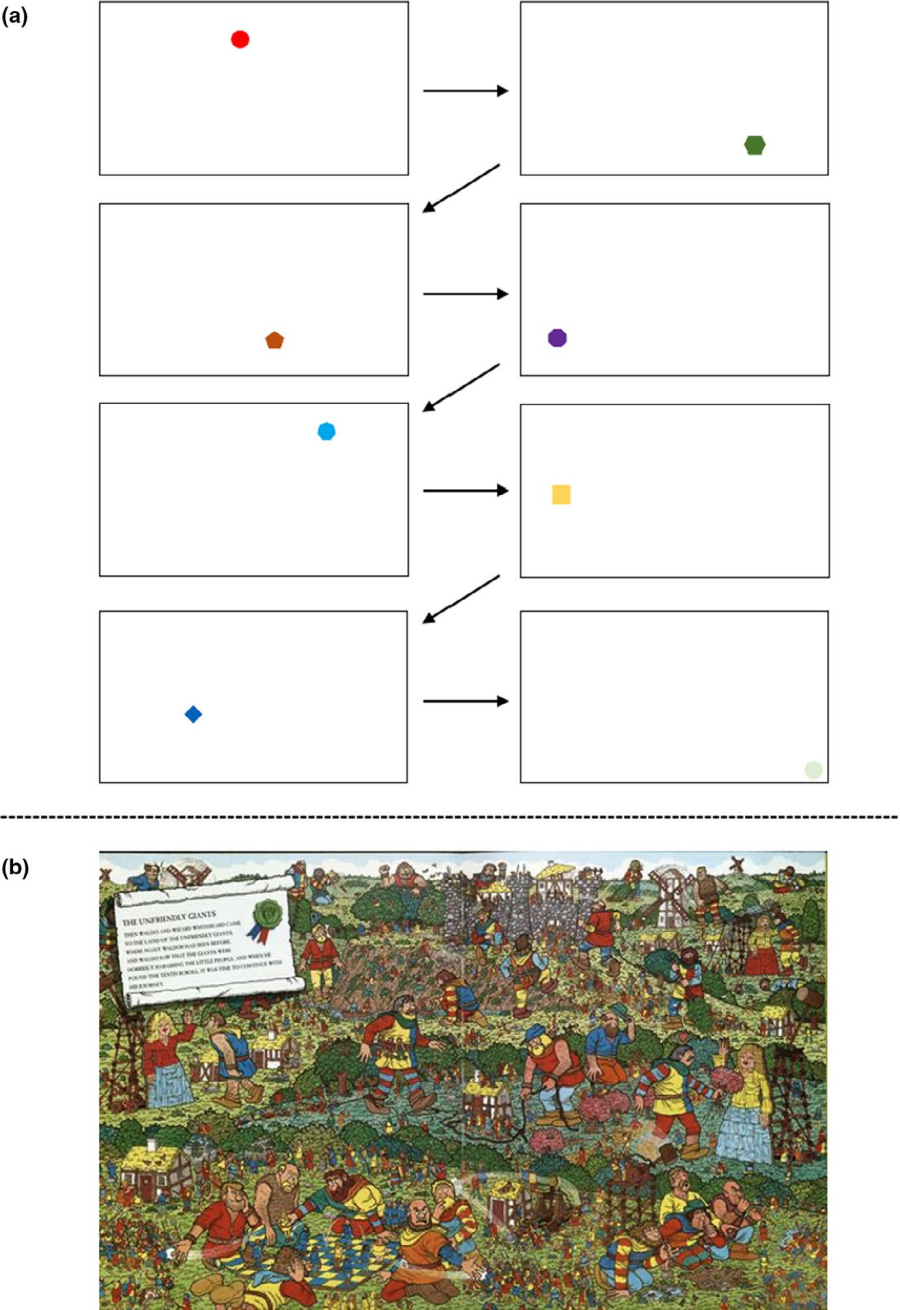
Representations of the two visual paradigm searches (a) the simple shape visual presentation and (b) the complex NVC task. The first three trials included viewing a simple visual presentation (a), where simple geometric shapes (i.e., circle, square, triangle, etc.) of different colors moved to randomized points across the visual display. These shapes changed positions at three different speeds: 4‐, 2‐, and 0.5‐s, where the order of the three NVC tasks was randomized prior to participant arrival. Additionally, it is important to note that this sequence is one example of how the geometric shapes were presented. However, as this presentation was randomized based on location, shape, and color; there is an endless possibility of potential shape sequences. The complex NVC task involved a complex visual search paradigm “*Where's Waldo?*” (b). Screen brightness was maximized, and the entire 27‐inch screen was utilized

All testing occurred over the course of a single laboratory visit in a highly controlled testing environment within the Cerebrovascular Concussion Laboratory at the University of Calgary. All testings were completed within the normal workday for all individuals, which previous research has shown NVC metrics are nominally impacted by diurnal variation during this time period (Burma et al., 2021). The elevation of the laboratory was 1,111 meter above sea level. The median and range across all testing sessions for environmental factors were: barometric pressure (median: 667.0 millimeters of mercury [mmHg], range: 662.3–678.5 mmHg), humidity (median: 19.9%, range: 14.0–61.9%), and temperature (median: 21.6°C, range: 20.1–22.4°C) (Table 1). Consistent with prior research, participants engaged in four different visual paradigm trials where they were seated 50–60 cm away from a 27‐inch Acer monitor (Smirl et al., 2016). The screen brightness was maximized, and the entire 27‐inch screen was utilized for the four visual tasks. If required, participants wore corrective eyewear or contacts to ensure they had 20/20 vision. The first three trials included viewing a simple visual presentation, where geometric shapes (i.e., circle, square, triangle, hexagon, etc.) 2 cm in size (along the longest axis) moved around the visual display and were randomized based on location, color, and shape (Figure 1). Shape position changed at three different durations: 4, 2, and 0.5 s. The fourth task involved a complex visual search paradigm (“*Where's Waldo?*”), of which has been previously utilized as it requires a large metabolic demand from the visual processing centers (Burma et al., 2021; Smirl et al., 2016). To best understand subjective task engagement, the randomization between tasks was completed in two different ways for half of the subjects. The first, included randomizing the order of the three simple shapes, concluding with “*Where's Waldo?*”. The second, included a true randomization of all four tasks. As the “*Where's Waldo?*” is a goal‐orientated task compared to the simple shapes, the researchers wanted to control for this difference by concluding with the “*Where's Waldo?*” task for half of the subjects. Nonetheless, no differences were noted in subjective task engagement between the first and second randomization protocols for each task and therefore, to maintain power, they were combined. The randomization of the NVC tasks was completed via a “Spin the Wheel – Random Picker” app (Kaunas, Lithuania, https://spinthewheel.app) prior to participant arrival. Additionally, as stated, the geometric shapes changed in color between each presentation to aid in stimulating the ventral stream in addition to the dorsal stream. The different duration presentations were used to represent different levels of engagement and visual demands within the visual processing centers. Previous research used a simpler version of the shape task, where the images were only presented in two locations with shape color remaining consistent (Smirl et al., 2016). The 4‐s duration was utilized as it represented a task involving low engagement and low cortical demand within the visual processing centers. The 2‐s task required moderate processing demands within the occipital regions of the brain and was classified as a low to moderately engaging task. The 0.5‐s duration was designed to represent a visual task that would elicit moderate to high levels of engagement and stimulation of the visual cortices. This speed was strategically chosen as the eye requires ~200 milliseconds (ms) to initiate a saccadic eye movement to an unexpected stimulus, and subsequently ~100–200 ms to correctly fixate the eyes upon a visual presentation (Rayner, 1998, 2009; Rayner & Raney, 1996). Therefore, a duration of 0.5‐s (i.e., 500 ms) was utilized to give the participants enough time to locate and fixate on each shape before a change in location occurred, similar to how one would scan a complex scene search paradigm. A benefit of using the “*Where's Waldo?*” paradigm is that it causes a robust and substantial physiological response within the vessel of interest (Smirl et al., 2016). This response greatly overcomes the naturally occurring physiological signals (i.e., Mayer waves, respiratory sinus arrhythmias, etc.), which explains why the task is considered to augment the signal (i.e., NVC response) to noise (i.e., naturally occurring physiological processes) ratio (Smirl et al., 2016). However, a limitation of this technique is the potential for increased activation in additional brain regions due to the fact “*Where's Waldo?*” requires object recognition/identification within the “*what*” stream in addition to other cortical structures (prefrontal cortex, parietal cortex, later geniculate nucleus, amygdala, etc.) (Chang et al., 2014). Therefore, the 0.5‐s protocol was used to understand the extent to which “*Where's Waldo?*” activates more than just the visual centers, as the eye movements made during the 0.5‐s task would resemble the rapid eye movements made during “*Where's Waldo?*”. Following each trial, participants were asked to rate how engaging they found each task on an ordinal scale ranging from 1 to 10. Participants were told a score of 1 was associated with a research task participants found extremely “unengaging”; whereas a score of 10 was associated with a research task that was the “most engaging.” While there is some limitation of using subjective scales (Jahedi & Méndez, 2014), this is the first investigation to see if a potential correlation exists between subjective engagement and the NVC response, which will help guide future investigations to understand the best methodological approach to assess NVC metrics.

**FIGURE 2:**
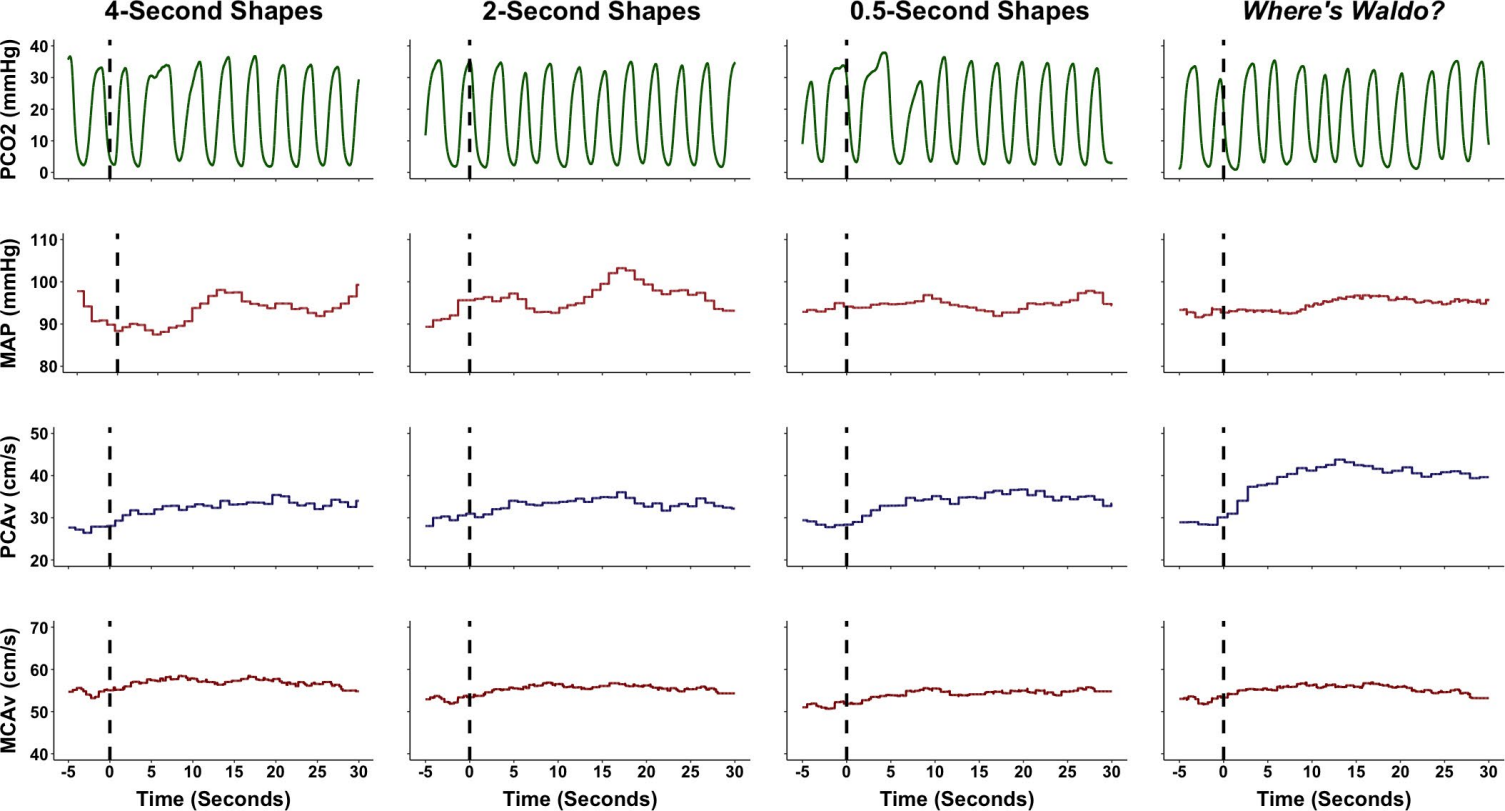
A representative trace from an individual during each of the four tasks. These included viewing simple geometric shapes of different colors that changed location on a screen at different speeds (i.e., 4‐, 2‐, and 0.5‐s) and a complex visual scene search “*Where's Waldo?*”. The dashed line signals the eyes‐open period where the participant begun to engage in each respective task. The 5‐s prior to the eyes‐open stimulus was utilized to determine the baseline eyes‐closed metrics; whereas the 30 seconds following the eyes‐open stimulus was used to quantify the neurovascular coupling response. The partial pressure of carbon dioxide (PCO2), millimeters of mercury (mmHg), mean arterial pressure (MAP), posterior cerebral artery blood velocity (PCAv), centimeter per second (cm/s), and middle cerebral artery blood velocity (MCAv)

Participants completed eight NVC cycles comprised of a minimum of 20‐s eyes‐closed, followed by 40‐s with eyes‐open to the visual task, in accordance with previous recommendations (Burma et al., 2021; Phillips et al., 2016; Smirl et al., 2016). During the “*Where's Waldo?*” task, participants were presented with a new puzzle for each of the eyes‐open trials. First, participants were instructed to attempt to locate Waldo within the puzzle; however, if located, participants were instructed to locate secondary characters (i.e., Wenda, Odlaw, Woof's Tail, and Wizard Whitebeard) (Handford & Little, 1987). None of the participants were able to find all five characters within the 40‐s, mitigating the likelihood any participant stopped engaging in the task before each NVC trial was completed. Moreover, the 20‐s eyes‐closed section was examined on a trial‐by‐trial basis and extended as necessary to ensure all physiological variables returned to baseline prior to asking the participant to open their eyes and engage in the task. This enhanced the signal‐to‐noise ratio, through mitigating against the augmentations in CBV would be influenced by other physiological factors (e.g., Mayer waves) (Julien, 2006). A 40‐s eyes‐open period ensured that maximal PCA and MCA velocities were reached. Peak CBV is commonly achieved at ~20–30‐s after the eyes‐open during complex scene search paradigms, the 40‐s eyes‐open protocol reduced human error surrounding the timing of stimulus withdrawal (Burma et al., 2021). A representative trace from an individual from one cycle of each task is displayed in Figure 2.

### 2.4 Instrumentation

Heart rate was determined using a 3‐lead electrocardiogram, sampling RR intervals (ADI Instruments, Colorado Springs, CO, USA). Beat‐to‐beat blood pressure was measured using finger photoplethysmography with a brachial cuff corrected to heart height (Finometer NOVA; Finapres Medical Systems, Amsterdam, The Netherlands) (Omboni et al., 1993; Sammons et al., 2007). A pre‐calibrated inline gas analyzer was used to assess breath‐to‐breath‐peak expired carbon dioxide to determine the end‐tidal partial pressure of carbon dioxide (P_ET_CO_2_) (ML206; AD Instruments). Deep vascular CBV in response to NVC tasks was assessed using TCD (DWL USA, Inc). Measures of TCD were sampled with two ultrasound probes at 2‐MHz, placed on the transtemporal windows to measure blood velocity in the MCA and PCA. The M1 segment of the MCA was insonated on the right side of the head, while the P1 segment of the PCA was insonated on the left. Vessels were located by experienced technicians through signal depth and CBV signal feedback to a finger tracking visual task and carotid compressions (Willie et al., 2014). Once the vessel location was confirmed, the probes were locked into place with a fitted head frame (DWL USA, Inc). All data were time‐aligned, collected, and stored using commercially available software (LabChart Pro Version 8, AD Instruments).

### 2.5 Data processing

Mean PCA blood velocity, MCA blood velocity, and mean arterial pressure were calculated using cyclic measurements in LabChart Pro employing the peak systolic and end diastolic values from each respective trace. Peak expired carbon dioxide on a breath‐to‐breath basis was used to derive P_ET_CO_2_ values. All channels were inspected for artifacts and filtered to remove outliers and noise, in an identical fashion for all participants. For example, during a trial, if a signal artifact was introduced into the data causing a massive millisecond spike in CBV, this was manually processed and changed to the correct CBV based on the surrounding values. Additionally, as this project was seeking to delineate the NVC response based on task, any trials that were visually confounded by Mayer waves, were removed from the individual subject averaged data to minimize the confounding influence of other physiological covariates. This took place in <5% of the overall dataset. All NVC trials were time‐aligned to the eyes‐open, stimulus onset, and were averaged for each participant within each respective task (i.e., 4, 2, 0.5 s, “*Where's Waldo?*”. The outcome measures of interest were baseline and peak CBV, relative percent increase in CBV, time‐to‐peak CBV increase, and the area under the curve during the first 30‐s of stimulus onset (AUC_30_; i.e., total activation) in both the PCA and MCA. The baseline CBV measures were averaged from the 5‐s immediately preceding the eyes‐open command. While the eyes‐closed protocol was ~20 s, the last 5‐s were used to ensure baseline CBV levels were reached prior to initiating the next trial. Peak CBV was determined as the maximum velocity achieved during the first 30‐s of engaging in each visual task. The relative percent increase in CBV was the change between baseline and peak CBV. Time‐to‐peak was the duration between the eyes‐open command and when peak CBV was achieved. Finally, total activation (AUC_30_) was calculated as the total area under the curve for the CBV response between baseline and the CBV response over the first 30‐s of the eyes‐open period. The distinction between AUC_30_ and the relative percent increase in CBV can be seen in Figure 2 in the prior work by Smirl et al. (2016) between the reading and colored dots task within the PCA trace. The reading task evoked an increase in PCAv that was elevated upon the participant opening their eyes, which remained at/near the peak value across the 30 s. However, with the colored dots task, the PCAv followed a similar increase within the first 5‐s compared to the reading task; however, PCAv was then attenuated for the next 20 s. Therefore, while the relative percent increase in CBV would have been somewhat similar between tasks, the AUC_30_ was drastically reduced, as it considers the CBV increase associated with the activation of the vasculature over the initial 30‐s of the task. This means AUC_30_/total activation may be more sensitive at revealing subtle alterations to the vasculature than just using the relative percent increase in CBV independently, that has been commonly employed in the broader NVC literature (Azevedo et al., 2007; Panerai et al., 2019; Willie et al., 2011). For further details regarding these variables, readers are directed to Figure 2 in Burma et al., (2021) where they are visually represented. Self‐written Excel scripts (Microsoft) were used to calculate the aforementioned variables.

### 2.6 Sample size calculation

G*power (v3.1.9) was used to determine the sample size required for the present investigation using data from a previous study performed by Smirl et al. (2016). The a priori power was calculated utilizing data from the area under the curve of the PCA between colored dots and the “*Where's Waldo?*” search. A sample size of six participants was required to achieve an alpha of 0.05, at a power greater than 95% (effect size = 2.23). However, as it was unknown if a 0.5‐second task would provide a sufficient stimulus to provoke a robust enough response compared to “*Where's Waldo?*”, different than the 4‐s and/or 2‐s task, the sample size was tripled to ensure adequate power to assess potential differences between all tasks and between biological sexes. This will help future studies understand the power required to delineate potential biological sex differences.

### 2.7 Statistical analyses

Data were analyzed using R‐Studio (version 1.3.1056) (R Core Team. R: A, 2020). Two‐factor repeated‐measures analysis of variance (ANOVA), with levels of task (i.e., 4‐s shapes, 2‐s shapes, 0.5‐s shapes, and “*Where's Waldo?*”) and biological sex (i.e., male and female) were calculated for each NVC variable of interest (Blanca et al., 2017). Previous research has demonstrated ANOVAs do not inflate the Type I error rate with unequal and equal group sizes or distribution of the data and regardless of total sample size greater than 15 participants (Blanca et al., 2017). Tukey post hoc comparisons were performed to delineate where the simple effect differs following a significant omnibus test result. For all ANOVAs, effect sizes were calculated through the generalized eta squared (η^2^ *_G_*) coefficient, as there has been growing concern about deriving interpretations independently from *p*‐values (Amrhein et al., 2019), especially within physiological/biomedical studies (Panagiotakos, 2008). Consistent with previous research, thresholds of 0.02, 0.13, and 0.26 were used to determine small, medium, and large effect sizes, respectively (Bakeman, 2005). Additionally, Cohen's *d* effect sizes were computed to determine the effect sizes for the post hoc, pairwise comparisons, where thresholds of <0.2, 0.2–0.5, 0.5–0.8, and >0.8 were used to delineate negligible, small, moderate, and large effect sizes, respectively (Lakens, 2013). For engagement ratings, a Kruskal–Wallis one‐way ANOVA compared engagement scores between tasks. If the Kruskal‐Wallis test produced a significant omnibus test, Wilcoxon signed‐ranked tests with Cliff's delta effect sizes were used to determine where a difference was between tasks. Additionally, Mann–Whitney U tests and calculated Cliff's delta effect sizes to compare between sexes for engagement scores. For Cliff's delta effect sizes, thresholds of <0.147, 0.147–0.3, 0.33–0.474, and >0.474 were utilized (Romano et al., 2006). As sample size has a greater influence on *p*‐values compared to effect size, both were considered when making inferences about the data and statistical analysis (Sullivan & Feinn, 2012). Spearman's rank correlation coefficient values (*ρ*) were computed to understand if there is a correlation between subjective ratings of task engagement with the NVC response for each outcome variable. The Spearman's coefficients were categorized as: 0.00–0.30 (negligible), 0.30–0.50 (low), 0.50–0.70 (moderate), 0.70–0.90 (high), and 0.90–1.00 (very high) (Mukaka, 2012). Finally, within‐subject coefficient of variation (CoV) values were calculated for all physiological parameters between the four tasks to understand the magnitude of variation between tasks, as physiological confounding is a threat to the internal validity of physiological studies. Consistent with previous research, CoV values for physiological research of <10% and <20% was considered good and acceptable, respectfully (Hopkins, 2000). The calcualted CoV metrics were displayed as estimate (95% confidence interval). The CoV value was calculated using a bootstrap approach with 10,000 resamples, where the mean from all participants was used to calculate the CoV estimates and associated 95% confidence intervals. Significance was determined a priori at *α* = 0.05 and corrected based on multiple comparisons. Data are displayed as mean ±95% confidence intervals for all NVC outcome metrics of interest (i.e., baseline and peak CBV, the percent increase in CBV, time‐to‐peak CBV, and AUC_30_).

## 3 RESULTS

### 3.1 Cardiovascular and respiratory parameters during the visual tasks

All cardiovascular and respiratory parameters during each of the tests are displayed in Table 2. A main effect for sex was present for mean arterial pressure (*F*_(1,148)_ = 5.88, *p* = 0.001, η^2^ *_G_* = 0.04 [small]) (Table 2). However, there were no sex main effects for P_ET_CO_2_ (*F*_(1,148)_ = 2.77, *p* = 0.098, η^2^ *_G_* = 0.02 [negligible]), respiratory rate (*F*_(1,148)_ = 0.77, *p* = 0.383, η^2^ *_G_* = 0.01 [negligible]), or heart rate (*F*_(1,148)_ = 0.03, *p* = 0.869, η^2^ *_G_* = 0.01 [negligible]) (Table 2). Moreover, there were no task or task‐by‐sex main effects for any cardiovascular and respiratory parameters (all *F*_(3,148)_ < 0.25, all *p* > 0.862, all η^2^ *_G_* ≤ 0.02 [negligible]). Finally, the point estimates and 95% confidence intervals for all physiological variables were <10%, demonstrating the high level of physiological control across the four conditions for each participant (Table 2).

**TABLE 2:** Cardiovascular and respiratory variables across all conditions in 39 individuals (22 females)

	4 s	2 s	0.5 s	Waldo	Between task CoV (95% CI)
P_ET_CO2 (mmHg)	34.4 ± 5.3	34.4 ± 5.2	34.2 ± 5.0	34.3 ± 5.3	2.6% (2.1–3.1%)
Female	33.8 ± 5.2	33.6 ± 5.1	33.6 ± 4.6	33.9 ± 4.9	2.8% (2.2–3.5%)
Male	35.2 ± 5.5	35.5 ± 5.3	35.0 ± 5.6	34.8 ± 5.8	2.3% (1.6–3.0%)
Respiratory rate (BPM)	13.5 ± 3.3	13.6 ± 3.3	13.8 ± 3.5	13.7 ± 3.5	6.3% (5.3–7.2%)
Female	13.2 ± 3.2	13.3 ± 3.1	13.9 ± 3.7	13.3 ± 3.4	7.1% (5.9–8.3%)
Male	13.8 ± 3.5	13.9 ± 3.7	13.7 ± 3.4	14.3 ± 3.6	5.3% (4.1–6.5%)
Mean arterial pressure (mmHg)*	86.7 ± 13.1	86.7 ± 13.1	86.2 ± 13.4	87.8 ± 14.8	4.6% (3.9–5.3%)
Female	84.9 ± 13.8	85.4 ± 14.0	82.9 ± 12.7	85.7 ± 17.2	5.4% (4.5–6.3%)
Male	89.2 ± 12.1	89.9 ± 11.0	90.5 ± 13.4	90.4 ± 10.8	3.4% (2.7–4.2%)
Heart rate (bpm)	75.3 ± 12.4	75.3 ± 12.4	74.3 ± 12.2	73.3 ± 12.0	3.2% (2.6–3.8%)
Female	75.4 ± 10.9	75.5 ± 10.0	74.4 ± 9.2	73.6 ± 10.3	3.2% (2.4–4.0%)
Male	75.3 ± 14.5	75.3 ± 15.3	74.1 ± 15.7	72.9 ± 14.2	3.1% (2.2–3.9%)

Values are mean ± standard deviation. End tidal values of carbon dioxide (P_ET_CO_2_), respiratory rate (RR), breaths per minute (BPM), millimeters of mercury (mmHg), and beats per minute (bpm). The asterisk (*) denotes mean arterial pressure differed between sexes across all tasks (*p* < 0.001), albeit with a small effect size (Cohen's *d* = 0.40). The coefficient of variation (CoV) values were calculated using the mean values from each subject, where a bootstrap approach with 10,000 resamples was utilized to determine the associated 95% confidence intervals (CIs).

### 3.2 Correlations between task engagement and neurovascular coupling response

**FIGURE 3:**
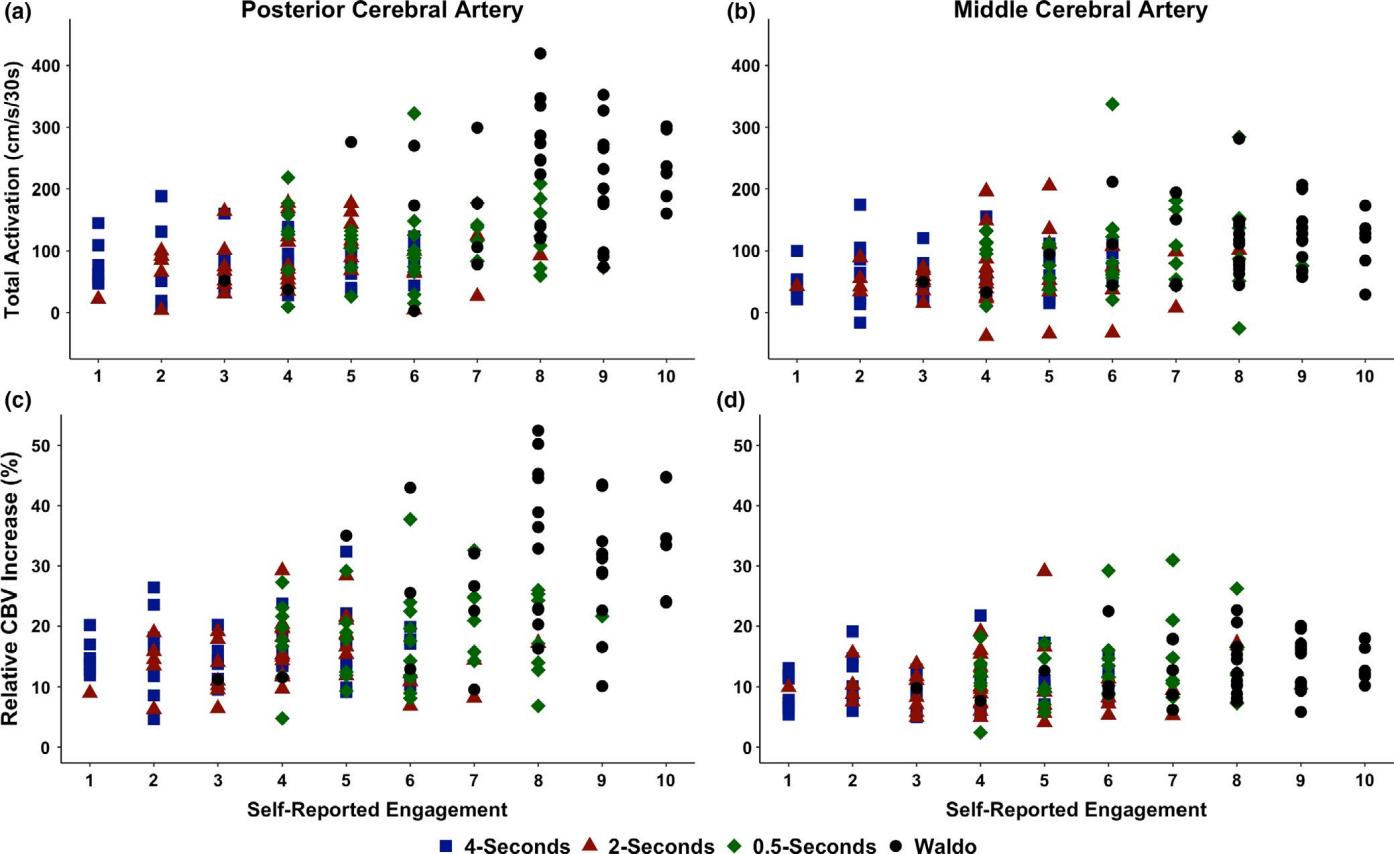
The Spearman's rank correlation between self‐reported task engagement and total activation/the area under the curve during the first 30‐s of stimulus (AUC_30_) within the (a) posterior cerebral artery (PCA) and (b) middle cerebral artery (MCA) and the relative percent (%) increase in CBV blood velocity from the eyes‐closed to eyes‐open stimulus within the (c) PCA and (d) MCA in 39 individuals (22 females and 17 males). Centimeter per second per 30‐seconds (cm/s/30s)

The median and range values for subjective ratings of task engagement, as well as the correlations between subjective task engagement and the NVC response for each variable, stratified between sexes are displayed in Table 3. A difference was found between tasks for subjective levels of task engagement (*H*_(6)_ = 7923, *p* < 0.001). The post hoc analysis revealed engagement was different between all tasks (all *p* < 0.001, all Cliff's delta > 0.60 [large]), aside from the 4‐ versus 2‐s comparison (*p* = 0.065, Cliff's delta = 0.24 [small]) (Table 3). Furthermore, no differences were present between males and females with respect to subjective levels of engagement for each task: 4‐s (*p* = 0.085, Cliff's delta = 0.32 [small]); 2‐s (*p* > 0.772, Cliff's delta = 0.05 [negligible]); 0.5‐s (*p* = 0.219, Cliff's delta = 0.32 [small]); and Waldo (*p* = 0.651, Cliff's delta = 0.09 [negligible]) (Table 3). Moreover, the most relevant markers of the NVC response (i.e., AUC_30_ and the relative percent increase in CBV) showed a greater correlation among males compared to their female counterparts (Table 3). Additionally, as total activation (AUC_30_) and the relative percent increase are widely used to quantify the NVC response, the data points for each of these metrics were plotted against the subjective ratings of task engagement (Figure 3).

**TABLE 3:** Subjective ratings of task engagement and corresponding Spearman's rank correlation coefficient (ρ) during neurovascular coupling assessments within the Posterior Cerebral Artery (PCA) and the Middle Cerebral Artery (MCA)

	Total (n = 39)	Females (n = 22)	Males (n = 17)
Subjective task engagement ratings			
4‐s	3 (2–4)	4 (1–4)	3 (1–3)
2‐s	4 (3–5)	4 (3–5)	4 (3–5)
0.5‐s	6 (5–8)	6 (5–8)	6 (5–8)
Waldo	8 (7–9)	9 (8–9)	8 (7–9)
Neurovascular coupling variable			
PCA baseline	*p* = 0.254; *ρ* = 0.09	*p* = 0.568; *ρ* = 0.06	*p* = 0.715; *ρ* = 0.05
PCA peak	*p* < 0.001; *ρ* = 0.30	*p* = 0.016; *ρ* = 0.26	*p* = 0.004; *ρ* = 0.34
PCA percent increase	*p* < 0.001; *ρ* = 0.48	*p* < 0.001; *ρ* = 0.38	*p* < 0.001; *ρ* = 0.60
PCA AUC_30_	*p* < 0.001; *ρ* = 0.51	*p* < 0.001; *ρ* = 0.43	*p* < 0.001; *ρ* = 0.58
PCA time‐to‐peak	*p* = 0.002; *ρ* = 0.26	*p* < 0.001; *ρ* = 0.36	*p* = 0.121; *ρ* = 0.19
MCA baseline	*p* = 0.002; *ρ* = 0.24	*p* = 0.007; *ρ* = 0.29	*p* = 0.149; *ρ* = 0.18
MCA peak	*p* < 0.001; *ρ* = 0.29	*p* = 0.002; *ρ* = 0.32	*p* = 0.036; *ρ* = 0.25
MCA percent increase	*p* < 0.001; *ρ* = 0.27	*p* = 0.010; *ρ* = 0.27	*p* = 0.006; *ρ* = 0.33
MCA AUC_30_	*p* < 0.001; *ρ* = 0.40	*p* < 0.001; *ρ* = 0.39	*p* < 0.001; *ρ* = 0.42
MCA time‐to‐peak	*p* = 0.267; *ρ* = 0.09	*p* = 0.197; *ρ* = 0.14	*p* = 0.485; *ρ* = 0.09

Values are medians (interquartile ranges). At the end of each task, the participants were asked to subjectively rate how engaging they found each task using an ordinal scale ranging from 1 to 10. A score of 1 was associated with an unengaging research task and a score of 10 was associated with a highly engaging research task. Aside from the comparison between 4‐ and 2‐s (*p* = 0.065), all subjective task engagement ratings were different (all *p* < 0.001; all Cliff's delta>0.60 [large]).

### 3.3 Posterior cerebral artery task, sex, and task‐by‐sex main effects

**FIGURE 4:**
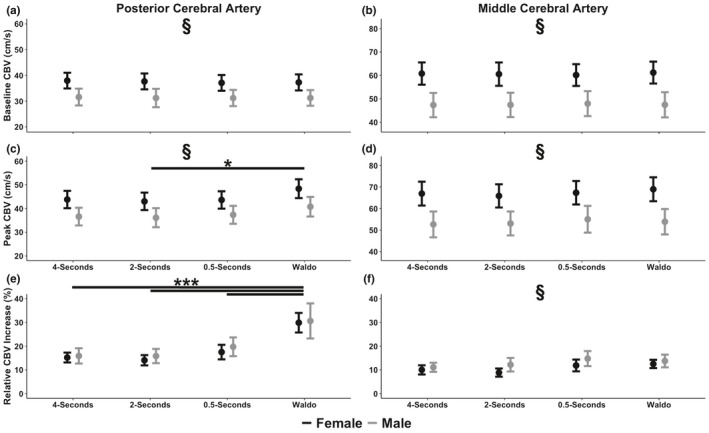
Mean ± 95% confidence intervals of (a) baseline posterior cerebral artery (PCA) blood velocity, (b) baseline middle cerebral artery (MCA) blood velocity, (c) peak PCA blood velocity,(d) peak MCA blood velocity, (e) the relative percent (%) increase in PCA blood velocity from the eyes‐closed to eyes‐open stimulus, and (f) the relative percent (%) increase in MCA blood velocity from the eyes‐closed to eyes‐open stimulus in 39 individuals stratified by biological sex (22 females and 17 males). Baseline measures were calculated during the 5‐s preceding the eyes‐open stimulation, whereas peak metrics were calculated during the visual task. The section symbol (§) denotes a NVC variable that differed between biological sexes (*p* < 0.05). The asterisk (*) denotes a difference between tasks at: *p* < 0.05 (*), *p* < 0.01 (**), and *p* < 0.001 (***). Centimeter per second (cm/s)

**FIGURE 5:**
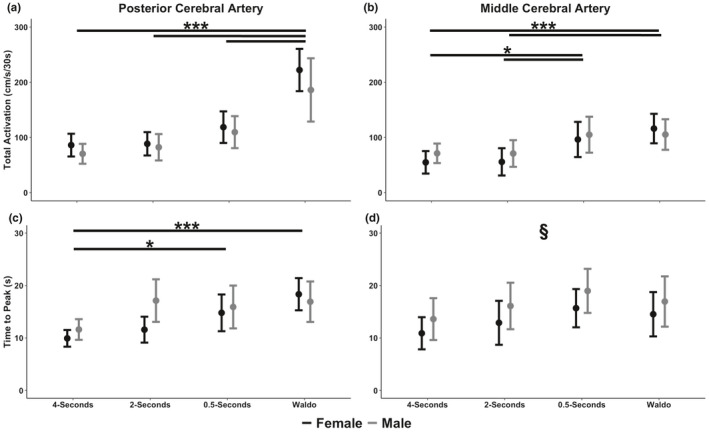
Mean ± 95% confidence intervals of total activation/the area under the curve during the first 30‐s of stimulus (AUC_30_) within the (a) posterior cerebral artery (PCA) and (b) middle cerebral artery (MCA) and the time‐to‐peak blood velocity in the (c) PCA and (d) MCA in 39 individuals stratified by sex (22 females and 17 males). The section symbol (§) denotes a NVC variable that differed between biological sexes (*p* < 0.05). The asterisk (*) denotes a difference between tasks at: *p* < 0.05 (*), *p* < 0.01 (**), and *p* < 0.001 (***). Centimeter per second per 30‐seconds (cm/s/30s)

A sex main effect was found for baseline PCA velocity (*F*_(1,148)_ = 32.9, *p* < 0.001, η^2^ *_G_* = 0.18 [moderate]); however, no task (*F*_(3,148)_ = 0.07, *p* = 0.974, η^2^ *_G_* = 0.001 [negligible]) or task‐by‐sex interaction (*F*_(3,148)_ = 0.02, *p* = 0.997, η^2^ *_G_* = 0.001 [negligible]) main effects were present (Figure 4a). Both sex (*F*_(1,148)_ = 28.5, *p* < 0.001, η^2^ *_G_* = 0.16 [moderate]) and task (*F*_(3,148)_ = 3.12, *p* = 0.028, η^2^ *_G_* = 0.06 [small]) main effects were found for peak PCA velocity, whereas no task‐by‐sex interaction (*F*_(3,148)_ = 0.05, *p* = 0.987, η^2^ *_G_* = 0.01 [negligible]) main effect was present (Figure 4c). A task (*F*_(3,148)_ = 31.9, *p* < 0.001, η^2^ *_G_* = 0.39 [large]) main effect was found for the relative percent increase in PCA velocity, but no sex (F_(1,148)_ = 1.14, *p* = 0.287, η^2^ *_G_* = 0.01 [negligible]) or task‐by‐sex (*F*_(3,148)_ = 0.09, *p* = 0.966, η^2^ *_G_* = 0.01 [negligible]) interaction main effects were present (Figure 4e). For PCA AUC_30_, a task main effect was found (*F*_(3,148)_ = 31.0, *p* < 0.001, η^2^ *_G_* = 0.39 [large]); however, there was no sex (*F*_(1,148)_ = 2.50, *p* = 0.116, η^2^ *_G_* = 0.02 [negligible]) or task‐by‐sex interaction main effect (*F*_(3,148)_ = 0.40, *p* = 0.75, η^2^ *_G_* = 0.01 [negligible]) (Figure 5a). A task main effect was present for time‐to‐peak PCA velocity (*F*_(3,148)_ = 7.80, *p* < 0.001, η^2^ *_G_* = 0.14 [small]) but no sex (*F*_(1,148)_ = 2.66, *p* = 0.105, η^2^ *_G_* = 0.02 [negligible]) or task‐by‐sex interaction main effects (*F*_(3,148)_ = 1.84, *p* = 0.142, η^2^ *_G_* = 0.04 [small]) were found (Figure 5c).

### 3.4 Middle cerebral artery task, sex, and task‐by‐sex main effects

A sex (*F*_(1,148)_ = 59.5, *p* < 0.001, η^2^ *_G_* = 0.29 [large]) main effect was found for baseline MCA velocity but not a task (*F*_(3,148)_ = 0.01, *p* = 0.998, η^2^ *_G_* = 0.01 [negligible]) or task‐by‐sex interaction main effect (*F*_(3,148)_ = 0.04, *p* = 0.990, η^2^ *_G_* = 0.01 [negligible]) (Figure 4b). Similarly, a sex (*F*_(1,148)_ = 49.4, *p* < 0.001, η^2^ *_G_* = 0.25 [moderate]) main effect was found for peak MCA velocity but not a task (*F*_(3,148)_ = 0.27, *p* = 0.847, η^2^ *_G_* = 0.01 [negligible]) or task‐by‐sex interaction main effect (*F*_(3,148)_ = 0.11, *p* = 0.954, η^2^ *_G_* = 0.01 [negligible]) (Figure 4d). Furthermore, a task (*F*_(3,148)_ = 4.13, *p* = 0.008, η^2^ *_G_* = 0.08 [small]) and sex (*F*_(1,148)_ = 7.59, *p* = , η^2^ *_G_* = 0.05 [small]) main effects were present, but no task‐by‐sex interaction main effect (*F*_(3,148)_ = 0.31, *p* = 0.820, η^2^ *_G_* = 0.01 [negligible]) for the relative percent increase in MCA velocity (Figure 4f). A task main effect (*F*_(3,148)_ = 8.21, *p* < 0.001, η^2^ *_G_* = 0.14 [moderate]) was found for MCA AUC_30_; however, no sex (*F*_(1,148)_ = 0.66, *p* = 0.416, η^2^ *_G_* = 0.01 [negligible]) or task‐by‐sex interaction (*F*_(3,148)_ = 0.488, *p* = 0.691, η^2^ *_G_* = 0.01 [negligible]) main effects were present (Figure 5b). For time‐to‐peak MCA velocity, a sex main effect was found (*F*_(1,148)_ = 4.44, *p* = 0.037, η^2^ *_G_* = 0.03 [small]) but no task (*F*_(1,148)_ = 2.43, *p* = 0.068, η^2^ *_G_* = 0.05 [small]) or task‐by‐sex interaction (*F*_(3,148)_ = 0.02, *p* = 0.995, η^2^ *_G_* = 0.01 [negligible]) main effects (Figure 5d).

### 3.5 Posterior cerebral artery neurovascular coupling pairwise comparisons

Females had a greater baseline PCA velocity (*p* < 0.001, Cohen's *d* = 0.95 [large]) (Figure 4a) and peak PCA velocity (*p* < 0.001, Cohen's *d* = 0.80 [large]) (Figure 4c) compared to males. Additionally, peak PCA velocity was greater for the “*Where's Waldo?*” task compared to the 2‐s task (*p* = 0.035, Cohen's *d* = 0.56 [moderate]), but not between any of the other tasks (*p *> 0.110, Cohen's *d *< 0.48 [negligible/small]) (Figure 4c). For the relative percent increase within the PCA, the Waldo task was greater than the 4‐s (*p* < 0.001, Cohen's *d* = 1.62 [large]), 2‐s (*p* < 0.001, Cohen's *d* = 1.70 [large]), and 0.5‐s tasks (*p* < 0.001, Cohen's *d* = 1.21 [large]) (Figure 4e). However, there were no differences between the 4‐ and 2‐s tasks (*p* = 0.982, Cohen's *d* = 0.12 [negligible]), 4‐s and 0.5‐s tasks (*p* = 0.345, Cohen's *d* = 0.47 [small]), and 2‐s and 0.5‐s tasks (*p* = 0.178, Cohen's *d* = 0.57 [moderate]) (Figure 4e). The post hoc test revealed the “*Where's Waldo?*” task elicited a larger PCA AUC_30_ compared to the 4‐s task (*p* < 0.001, Cohen's *d* = 1.68 [large]), 2‐s task (*p* < 0.001, Cohen's *d* = 1.57 [large]), and 0.5‐s task (*p* < 0.001, Cohen's *d* = 1.12 [large]) (Figure 5a). However, no differences were noted for PCA AUC_30_ between the 4‐s and 2‐s tasks (*p* = 0.972, Cohen's *d* = 0.15 [negligible]), 4‐ and 0.5‐s tasks (*p* = 0.086, Cohen's *d* = 0.69 [moderate]), and 2‐ and 0.5‐s tasks (*p* = 0.215, Cohen's *d* = 0.54 [moderate]) (Figure 5a). Finally, the time‐to‐peak PCA velocity was lower for the 4‐s task compared to “*Where's Waldo?*” (*p* < 0.001, Cohen's *d* = 1.24 [large]) and the 0.5‐s task (*p* = 0.012, Cohen's *d* = 0.75 [moderate]) (Figure 4c). However, no differences were noted between 4‐ and 2‐s (*p* = *0*.*117*, Cohen's *d* = 0.58 [moderate]), 2‐ and 0.5‐s (*p* = 0.825, Cohen's *d* = 0.17 [negligible]), 2‐s and Waldo (*p* = 0.064, Cohen's *d* = 0.52 [moderate]), and 0.5 and Waldo (*p* = 0.105, Cohen's *d* = 0.33 [small]) (Figure 5c).

### 3.6 Middle cerebral artery neurovascular coupling pairwise comparisons

Compared to males, females had a greater baseline MCA velocity across all tasks (*p* < 0.001, Cohen's *d* = 1.27 [large]) (Figure 4b). Likewise, females had a greater peak MCA velocity across all tasks (*p* < 0.001, Cohen's *d* = 1.16 [large]) (Figure 4d). The post hoc revealed a greater relative percent increase in MCA velocity in males compared to females (*p* < 0.001), albeit a small effect size (Cohen's *d* = 0.43) (Figure 4f). Furthermore, while the post hoc revealed no differences between any tasks regarding the relative percent increase in MCA velocity, numerous of these comparisons had a moderate effect sizes: 4‐ versus 0.5‐s (*p* = *0*.*072*, Cohen's *d* = 0.60), 4‐s versus Waldo (*p* = 0.086, Cohen's *d* = 0.52), 2‐ versus 0.5‐s (*p* = *0*.*051*, Cohen's *d* = 0.58), and 2‐s and Waldo (*p* = 0.061, Cohen's *d* = 0.53) (Figure 4f). However, negligible effect sizes were found between 4‐ versus 2‐s (*p* = 0.999, Cohen's *d* = 0.04) and 0.5‐s and Waldo (*p* > 0.999, Cohen's *d* = 0.02) (Figure 4f). Moreover, the “*Where's Waldo?*” task elicited a larger MCA AUC_30_ compared to the 4‐s (*p* < 0.001, Cohen's *d* = 0.99 [large]) and 2‐s tasks (*p* < 0.001, Cohen's *d* = 0.90 [large]) (Figure 5b). Likewise, the 0.5‐s task elevated the MCA AUC_30_ compared to the 4‐s (*p* = 0.016, Cohen's *d* = 0.68 [moderate]) and 2‐s tasks (*p* = 0.017, Cohen's *d* = 0.63 [large]) (Figure 5b). However, no differences were noted between the 4‐s and 2‐s tasks (*p* > 0.999, Cohen's *d* = 0.01 [negligible]) and between the 0.5‐s and Waldo tasks (*p* = 0.808, Cohen's *d* = 0.18 [negligible]) (Figure 5b). Finally, time‐to‐peak MCA velocity was increased in males compared to females (*p* = 0.037), albeit with a small effect size (Cohen's *d* = 0.34) (Figure 5d).

## 4 DISCUSSION

The present study examined how image duration and visual complexity influence the NVC response in both the PCA and MCA, while also investigating how this response is impacted by biological sex and correlated with self‐reported subjective ratings of engagement. The key findings from this study were: (a) when comparing the simple visual searches, no difference in the PCA CBV response was found between the quickest search task (0.5‐s intervals) compared to the 2‐ and 4‐s paradigms; (b) the complex visual search task (“*Where's Waldo?*”) invoked the greatest CBV response in the PCA supplied regions of the brain when compared to all simple shape tasks; (c) the NVC response within the MCA was greater for both the “*Where's Waldo?*” and the simple shapes 0.5‐s task compared to the 2‐ and 4‐s tasks; (d) self‐reported measures of engagement displayed moderate correlations with CBV responses in total activation (i.e., AUC_30_) PCA velocity; however, the correlation between task engagement and total activation and the relative percent increase in PCA velocity was greater in males compared to females; and (e) females had a greater absolute baseline and peak CBV; however, had a similar NVC response compared to males.

### 4.1 Comparison with previous studies

Previous research has demonstrated the temporal NVC response can be effectively assessed through TCD by the implementation of various visual paradigms to stimulate a CBV response within the PCA (Gommer et al., 2014; Smirl et al., 2016; Willie et al., 2011). It has also been demonstrated that simple visual tasks evoke a moderate CBV response compared to more complex visual paradigms, such as “*Where's Waldo?*” (Smirl et al., 2016). Previous results are consistent with this study, as the “*Where's Waldo?*” visual paradigm evoked the most robust response in the PCA when compared to simpler visual tasks with a range (0.5–4 s) of image duration (Figures 4 and 5). Additionally, it has been suggested during visual processing, the MCA supplied regions of the cortices are activated to a lesser extent than the PCA supplied regions of the cortices (Büchel & Friston, 1997; Gitelman et al., 2002). Current findings mirror this proposition, as the NVC response was lower within the MCA compared to the PCA; however, a novel finding in this investigation is the fact the MCA response was increased in response to both the “*Where's Waldo?*” task and the 0.5‐s simple shapes tasks, compared to both the 2‐ and 4‐s tasks (Figure 4F). Ultimately, these findings highlight that the “*Where's Waldo?*” task elicited the most robust NVC response within the PCA compared to simple shapes tasks. Additionally, while this complex task also increased the MCA NVC response, this was not different compared to the 0.5‐s task, indicating the activation within the MCA may be due to the rapid eye movements made during these tasks. Moreover, the findings of the augmented MCA response could also be attributed to the elevated cognitive processing associated with the elevated cognitive demands of both the 0.5‐s task and the “*Where's Waldo?*” task and/or the integration of both the ventral “*what*” stream and the dorsal “*where*” stream. It is important to highlight that previous research has suggested a visual NVC response, should elicit a ~8%–10% relative increase in MCA velocity (Phillips et al., 2016). While the MCA percent increase was slightly higher within the “*Where's Waldo?*” and 0.5‐s tasks (~13–14%), the MCA percent increase was ~10%–12% during the 2‐ and 4‐s simple shapes tasks. Therefore, while the 4‐s task was designed to be minimally engaging and of low complexity, these results demonstrate that regardless of the visual task utilized, other areas of the cortex will be activated.

This was the first investigation to explore if subjective task engagement plays an underappreciated role in the NVC response. It was found task engagement was moderately correlated with the NVC response, which showed a greater correlation for males compared to their female counterparts (Figure 3 and Table 3). Therefore, future studies should take this finding into consideration when developing their methodology, utilizing the most complex task in order to quantify the NVC response. Finally, in concordance with previous research demonstrating females have greater absolute resting cerebral blood flow/perfusion (Bertsch et al., 2009; Devous et al., 1986; Lu et al., 2011; Rodriguez et al., 1988), females showed a greater absolute baseline and peak MCA and PCA response across all tasks (Figure 4). However, no sex differences were noted between males and females, within AUC_30_ or the relative percent increase in CBV (Figures 4 and 5).

### 4.2 Physiological underpinnings between tasks

The suggested physiological mechanisms to describe the aforementioned differential CBV response are oculomotor control, engagement, and cortical processing (Chang et al., 2014; Gitelman et al., 2002; Rayner, 1998, 2009; Rayner & Raney, 1996). The complex visual search consists of many different objects, shapes, and colors that exist in the visual field surrounding the object of interest. Because of the diversity in the presented stimuli, both the ventral (“*what*”) and dorsal (“*where*”) streams of visual perception are activated to a higher level compared to the simple tasks (Chang et al., 2014). This model of cortical processing supports the results of this study, as PCA activation increased with the level of processing required by the PCA supplied regions of the cortices (Chang et al., 2014; Port et al., 2016; Smith & Henderson, 2011). The oculomotor control theory suggests saccades and fixation times occur in response to changes in visual paradigm complexity (Rayner, 1998, 2009; Rayner & Raney, 1996; Smirl et al., 2016). Furthermore, it has been stated this theory aids in elucidating the elevated CBV response to the complex visual paradigm, due to the increased fixation points and evoked saccade sizes (Bertera & Rayner, 2000; Rayner, 1998, 2009; Rayner & Raney, 1996). While this study was unable to track saccadic eye movements, it nonetheless investigated the CBV response to differing eyes movements and fixation rates as a way to evaluate this model. The current findings suggest that when the complex visual paradigm (“*Where's Waldo?*”) is compared to simple visual searches (with durations designed to be indicative of similar fixation rates and saccade sizes (Rayner, 1998, 2009; Rayner & Raney, 1996)), the NVC response evoked by the complex paradigm within the PCA was more robust (Figures 4 and 5). Moreover, the regions of the brain involved with eye movements, which are supplied by the MCA, were elevated with both the complex and simple shapes tasks at 0.5‐s providing support for the oculomotor control theory. Furthermore, the present investigation also wanted to understand if task engagement could help explain potential confounds for the NVC response, as levels of task engagement have also been proposed as a mechanism to stimulate an increased number of saccades (Gitelman et al., 2002). The current findings indicate self‐reported levels of engagement displayed moderate correlations with the PCA NVC response (Table 3 and Figure 3), which proposes the idea participant engagement levels with respect to the visual stimuli tasks presented may play a role in the elevated NVC response. This finding was especially paramount for males compared to females (Table 3).

### 4.3 Neurovascular coupling sex differences

Many researchers have postulated females display greater CBV than males (Bertsch et al., 2009; Devous et al., 1986; Lu et al., 2011; Rodriguez et al., 1988). Specifically, in research involving the use of TCD to evaluate the NVC response, baseline and peak CBV velocities, along with absolute cerebrovascular reactivity, have been found to be higher in females compared to males (Tallon et al., 2020). Several mechanisms for these variances have been suggested, such as hormone, hemoglobin, and cerebral metabolism differences (Lu et al., 2011; Tallon et al., 2020; Yamaguchi et al., 1986). Previous literature has proposed that for similar levels of oxygen to be delivered to the brain in females compared to males, females must have a higher CBV to compensate for lower hematocrit levels (Lu et al., 2011; Shaw et al., 1984). Other literature has stated women have higher cerebral metabolic rates of glucose due to the hormonal effects of estrogen (Leenders et al., 1990; Marchal et al., 1992; Yamaguchi et al., 1986). This study evaluated sex differences in the NVC response between males and females in response to different visual paradigms. Harmonious to previous reports, females displayed greater absolute CBV within the PCA and MCA during the eyes‐closed (baseline) and eyes‐open (peak) period (Figure 4). However, no differences were noted between sexes for PCA or MCA AUC_30_ and the relative percent increase in PCA velocity (Figures 4 and 5), but a sex difference was found for the relative percent increase in MCA velocity. However, the latter had a small effect size (Cohen's *d* = 0.43). Collectively, despite males and females having different starting points for the NVC response, due to differences in absolute baseline CBV values, it appears the NVC response in and of itself is not different between biological sexes. As total activation (AUC_30_) and the relative percent increase are dependent on the absolute values, for these to be the same, it would require females to have a larger increase in peak CBV than males. For example, as females had baseline values of ~37cm/s and males had baseline values of ~31cm/s, to achieve a relative percent increase, this would require an elevation of ~11cm/s and 9cm/s, respectively. Hence, the elevation in cerebral metabolic rates of glucose in females (Leenders et al., 1990; Marchal et al., 1992; Yamaguchi et al., 1986) may potentially explain why there were no differences in the AUC_30_ or relative percent increase in CBV. Nonetheless, future research should continue to explore biological sex differences across the various regulatory mechanisms in the brain.

### 4.4 Implications for future research

Employing eye tracking measures to objectively quantify the movements while utilizing the “*Where's Waldo?*” paradigm could provide future objective measures of saccades, eye movements, and fixation times to inform theories which further elucidate the robust NVC response evoked in response to complex visual stimulation. By implementing simple visual tasks that only elicit a mild CBV response, the relationship between neural activation and CBV increases in the PCA is underestimated (Smirl et al., 2016). For example, complex visual paradigms such as “*Where's Waldo?*” will evoke greater cortical activation and CBV compared to less complex tasks, such as viewing simple shapes in the primary vessel of interest (i.e., PCA) (Figures 4 and 5). It should also be noted that regardless of the visual task used, the MCA was also increased by 10%–15% across all tasks (Figure 4). This further exemplifies the utility of the “*Where's Waldo?*” task as it substantially augments the PCA response (~12%–15% greater), while only minimally elevating the MCA response (~0%–3% greater) (Figure 4).

**FIGURE 6:**
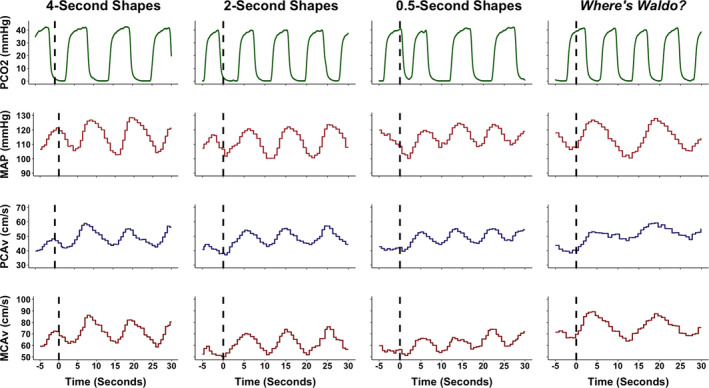
A representative trace from an individual with a high fitness level during each of the four tasks. The tasks included viewing simple geometric shapes of different colors that changed location on a screen at different speeds (i.e., 4, 2, and 0.5 s) and a complex visual scene search “*Where's Waldo?*”. The dashed line signals the eyes‐open period where the participant begun to engage in each respective task. The 5‐s prior to the eyes‐open stimulus was utilized to determine the baseline eyes‐closed metrics; whereas the 30‐s following the eyes‐open stimulus was used to quantify the neurovascular coupling response. As seen within the mean arterial pressure (MAP) trace, the individual has consistent Mayer waves that primarily impact the posterior cerebral artery blood velocity (PCAv) and middle cerebral artery blood velocity (MCAv) traces. However, from the data represented, only the “*Where's Waldo?*” evoked a sufficient neurovascular coupling (NVC) response to overcome the Mayer waves. This demonstrates in highly fit individuals; a simple visual task may be insufficient to properly assess the NVC response due to a low signal‐to‐noise ratio. Finally, it should be noted this individual is experiencing isolated systolic hypertension, which is common in athletes with a high‐to‐excellent fitness level. The partial pressure of carbon dioxide (PCO_2_), millimeters of mercury (mmHg), and mean arterial pressure (MAP)

As previously stated, NVC has been shown to be impaired following various clinical conditions (e.g., spinal cord injury (Phillips et al., 2014), traumatic brain injury (Hinzman et al., 2014), Alzheimer's disease (Nicolakakis & Hamel, 2011), etc.). Therefore, using a stimulus that is unlikely to elicit at least a modest response may limit clinical application and complicate the understanding of the physiological mechanisms underlying various diseases/disorders (Wright et al., 2017). As the “*Where's Waldo?*” and 0.5‐s tasks were shown to elicit activity in brain regions in addition to the occipital regions of the cortex (Figure 4), future research should utilize functional near‐infrared spectroscopy (fNIRS) to assess and accurately detect specific locations of cortical activation in the superficial layers of the brain (Csipo et al., 2021). It is suggested that fNIRS is capable of creating a cortical map with respect to specific regions engaged in an NVC task (Phillips et al., 2016). By utilizing these two measures (TCD and fNIRS) in a concurrent manner, future studies can objectively measure the deep and superficial blood supply to the cerebrum, in order to examine both the activation of the cortices and the corresponding CBV response. However, it should be noted previous TCD/fNIRS studies, while imperative, have been conducted independently rather than concurrently (Csipo et al., 2021). Anecdotally, a small subsample of individuals (n = 6) within the study had consistent and augmented Mayer waves present during all tasks (Figure 6). Among these participants, only the “*Where's Waldo?*” task provided a sufficient stimulus to overcome the naturally occurring physiological signals, further demonstrating its utility to quantify the NVC response by augmenting the natural signal‐to‐noise ratio. This is similar to cerebral autoregulatory assessments, where previous research has demonstrated that maximizing the signal‐to‐noise ratio, substantially increases both the validity and reliability for the outcome measures of interest (Burma et al., 2020; Claassen et al., 2009; Smirl et al., 2015).

### 4.5 Limitations

When utilizing TCD as an index of cerebral blood flow, the protocol assumes CBV is equivalent to cerebral blood flow as long as the diameter of the vessel observed is sustained (Ainslie & Hoiland, 2014). It has previously been demonstrated that when CO_2_ in the arterial blood (as indexed via P_ET_CO_2_) is within 8 mmHg of baseline values (eucapnia), the diameter of the arteries is thought to remain relatively constant (Ainslie & Hoiland, 2014). If P_ET_CO_2_ deviates more than 8 mmHg from eucapnia values, one cannot assume that the diameter of the vessel remained constant (Coverdale et al., 2014; Verbree et al., 2014). Therefore, P_ET_CO_2_ was closely monitored throughout the NVC trials in order to ensure CBV is an accurate index of cerebral blood flow. Further if required, participants were coached regarding breathing patterns to ensure P_ET_CO_2_ values remained similar across all four tasks and that differing breathing frequencies would not explain any differences between groups and/or sexes. Although these limitations are present, they likely had a minimal effect on the presented data as there was minimal variation in this measure between trials and sexes (Table 2). It should also be noted that hormone concentrations and phase of the menstrual cycle were not directly measured/controlled in this study. Therefore, future investigations should continue to explore if sex differences are present when these covariates are controlled for within the study design and/or analytical phase. Additionally, while the 0.5‐s presentation was designed to mimic the eye movements made during the “*Where's Waldo?*” paradigm, there would be a considerable difference in the manner in which the visual information was processed between the protocols. Nevertheless, this study was well designed to provide important and well‐powered exploratory insights on the complex relationship between metabolic demand and CBV in the deeper conduit vessels of the brain, while exploring the relationships between sex differences and subjective ratings of engagement with the NVC response. Finally, it should be noted there are limitations associated with using subjective scales (Jahedi & Méndez, 2014), specifically in regards to the reliability of the engagement scale to quantify individual perceptions of a task. Nonetheless, the integration of a rating of engagement was a novel consideration that future research should build upon to determine how this is correlated with the NVC response.

## 5 CONCLUSION

Consistent with prior literature, the complex visual scene search paradigm evoked the largest PCA NVC response when compared to simple paradigms designed to elicit similar saccade and fixation duration periods. Additionally, it was found that task engagement displayed a greater correlation with total activation (AUC_30_) and the relative percent increase in CBV within the PCA for males compared to females. Therefore, future studies quantifying NVC within the blood vessels and/or conduit vessels supplying the visual centers, should use a maximally engaging task, especially within males. Furthermore, females had greater absolute CBV levels during the eyes‐closed and eyes‐open periods; however, both sexes had similar NVC responses across all tasks. Conclusively, based on the current findings in conjunction with prior research, it is recommended complex visual search such as “*Where's Waldo?*” should be employed as a paradigm to evoke robust changes within the PCA in order to further elucidate cerebrovascular function within both healthy and clinical populations.

## CONFLICTS OF INTEREST

The authors declare they have no conflicts of interest.
